# Thermally Activated Swelling and Wetting Transition of Frozen Polymer Brushes:a New Concept for Surface Functionalization

**DOI:** 10.1002/adma.202502173

**Published:** 2025-04-14

**Authors:** Luciana Buonaiuto, Sander Reuvekamp, Billura Shakhayeva, Enqing Liu, Franziska Neuhaus, Björn Braunschweig, Sissi de Beer, Frieder Mugele

**Affiliations:** ^1^ Physics of Complex Fluids, MESA+ Institute University of Twente PO box 217 Enschede 7500AE The Netherlands; ^2^ Department of Molecules & Materials MESA+ Institute University of Twente PO box 217 Enschede 7500AE The Netherlands; ^3^ Institute of Physical Chemistry and Center for Soft Nanoscience University of Münster Corrensstraße 28/30 48149 Münster Germany

**Keywords:** adaptive wetting, microscale patterning, polymer brushes, soft materials, thermal responsiveness

## Abstract

Functional polymer brush coatings have great potential for various industrial applications thanks to their ability to adapt to environmental stimuli, providing tunable surface properties. While existing approaches rely on polymer‐solvent interactions and their response to external stimuli, changes in the intrinsic physical properties of the polymer also play a critical role in modulating brush behavior. In this context, the melting transition of a semicrystalline oleophilic poly‐octadecylmethacrylate (P18MA) brush coating is shown to drive a swelling and wetting transition upon exposure to various liquid alkanes. The top surface of this polymer displays a somewhat higher melting temperature than the bulk, enabling separate control of the bulk‐driven swelling and surface‐driven wetting transitions. Laser‐induced heating enables reversible on‐demand activation of both transitions with micrometer lateral resolution. These findings suggest a new concept of polymer brush‐based functional surfaces that allow for controlled fluid transport via separately switchable surface barriers and bulk transport layers based on a suitable choice of polymer‐polymer and polymer‐solvent interactions.

## Graphical abstract

A novel approach is introduced that leverages polymer phase transitions to modulate brush behavior. The oleophilic bottle brush system exhibits two distinct melting transitions—bulk and surface—enabling a two‐stage swelling and wetting transition. These transitions can be controlled globally, or locally with a focused laser beam, offering a new strategy for designing adaptive surfaces based on intrinsic polymer properties.

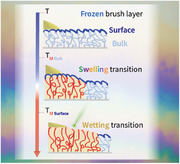

## 1 Introduction

Polymer brushes are used to modify the properties of solid surfaces for a variety of purposes^[^ ^1^, ^2^, ^3^ ^]^ including protection of the underlying material,^[^ ^4^ ^]^ improvement of biocompatibility,^[^ ^5^, ^6^ ^]^ adaptive control of droplet adhesion,^[^ ^7^, ^8^, ^9^, ^10^ ^]^ reduction of friction and adhesion,^[^ ^11^, ^12^, ^13^ ^]^ enhancement of selectivity and sensitivity in sensors^[^ ^14^, ^15^ ^]^ and in filtration and separation technologies.^[^ ^16^, ^17^ ^]^ Their versatility originates from their responsiveness to environmental stimuli. Chemically grafted at one end to the underlying substrate, polymer brushes can swell or collapse under the influence of external stimuli such as varying temperature and/or chemical composition of the ambient fluid. This modifies the interfacial properties and leads to the desired functionality. The design of polymer brush systems therefore typically focuses on optimizing the molecular interactions between the polymer and the solvent and/or specific solutes of interest. However, the physical properties of brush layers do not only change in response to variations of the polymer‐solvent interaction but also due to intrinsic transitions of the polymer such as melting and glass transitions.^[^ ^18^, ^19^, ^20^ ^]^ The transitions, from glassy to rubbery and liquid‐like behavior, affect solvent absorption,^[^ ^21^ ^]^ reduce roughness,^[^ ^18^, ^19^, ^20^ ^]^ and increase the polymer mobility.^[^ ^22^ ^]^ Thereby, they affect the wettability of the brushes^[^ ^7^, ^18^, ^19^, ^20^, ^23^ ^]^ and–more generally–offer an additional external handle to control and actuate the properties of the polymer brushes.

To explore the potential of this idea, we study the wetting properties of liquid *n*‐alkanes on oleophilic bottle brushes of poly‐*n*‐alkyl methacrylate (P*n*MA). The melting temperature of these polymer brushes can be tuned by varying the length of the side chains. Brushes with short side chains, such as P12MA are disordered and highly flexible at room temperature.^[^ ^24^ ^]^ They swell upon exposure to liquid alkanes, display low friction,^[^ ^25^ ^]^ and near‐complete wetting.^[^ ^26^ ^]^ In contrast, longer side chains promote crystallization both in the bulk^[^ ^24^, ^27^ ^]^ and at the surface.^[^ ^28^ ^]^ The richness of their intrinsic phase behavior makes this class of materials an ideal model system to investigate the impact of the polymer‐intrinsic melting transitions on the macroscopic swelling and wetting behavior. For P18MA, we show that the melting behavior of the polymer enables separate swelling and wetting transitions, which can be induced by global heating as well as local excitation with a focused laser beam. The resulting unique reversible control of wetting and solvent transport within the brush layer demonstrates the power of our conceptual approach, which should be generalizable to a broad range of materials and applications.

## 2 Results and Discussion

### 2.1 Swelling and Spreading Behavior

**Figure 1:**
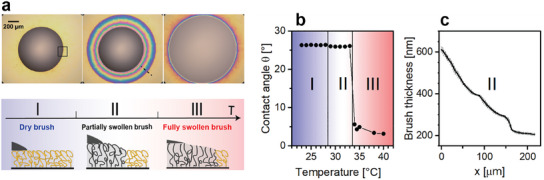
Temperature‐driven spreading and swelling dynamics of hexadecane on P18MA polymer brushes. a) Optical top‐view images and illustrative side‐view sketches (zoomed–in view of the region highlighted in the small box, top‐left image) of a hexadecane droplet (gray) on P18MA brushes (yellow). Left to right: regime I (*T* < 29 °C): partially wetting drop (θ = 26°) on solid dry brush; regime II (29°C < *T* < 33°C): partially wetting drop (θ = 26°) surrounded by halo of swollen brush; regime III (*T* > 33 °C): spreading drop (θ < 5°) on swollen brush. b) Contact angle versus temperature from side view images (Figure S1, Supporting Information) illustrating the invariance of the contact angle upon swelling (regime II) and the wetting transition upon entering regime III. c) Brush thickness profile along dashed line in panel a, regime II, as extracted from white light interferogram (see Experimental Section).

Oleophilic polymer brush layers of poly(octadecyl methacrylate) (P18MA) are prepared on a silicon wafer using a surface‐initiated atom transfer radical polymerization. The initial thickness in the dry, collapsed state ranges from 150 to 250nm, depending on the details of the synthesis (see ref. [26] and methods section). Drops of *n*‐alkanes deposited at room temperature onto such surfaces display a finite contact angle θ (≈26° for hexadecane). The color of the brush layer surrounding the drop and hence its thickness remain unaltered indicating that the frozen brush layer does not swell (regime I in **Figure** **1**). Upon increasing the temperature, we observe two distinct transitions. At the first transition from the low temperature regime I (room temperature) to the intermediate temperature regime II (29 °C for hexadecane), a colorful halo appears around the edge of the drop indicating local uptake of oil and swelling of the brush layer from its initial dry thickness (here: ≈230 nm) to more than 600 nm close to the contact line. This corresponds to a swelling ratio of ≈3 (Figure 1c). The contact angle, however, remains constant. At even higher temperatures (≈34 °C), regime III, the brush layer swells further and contact angle drops to values well below 5° (Figure 1b). The behavior remains consistent even after 15 consecutive temperature cycles without noticeable degradation, as shown in Figure S7 (Supporting Information). A similar sequence of swelling and wetting transition is observed for *n*‐alkanes with various chain lengths from dodecane (C12) to eicosane (C20). Yet, transition temperatures systematically shift toward lower transition temperatures for decreasing chain length (Videos S1–S5, Supporting Information). This two‐step swelling and wetting transition is strikingly different from the wetting of the same oils on bottle brushes with the same architecture but slightly shorter dodecyl side chains (P12MA), which has a melting temperature below room temperature. As a result, the chains of P12MA are very flexible and the brush exhibits pronounced swelling and near‐complete wetting (θ < 5°) even at room temperature.^[^ ^26^ ^]^

### 2.2 Nanomechanical Response

**Figure 2:**
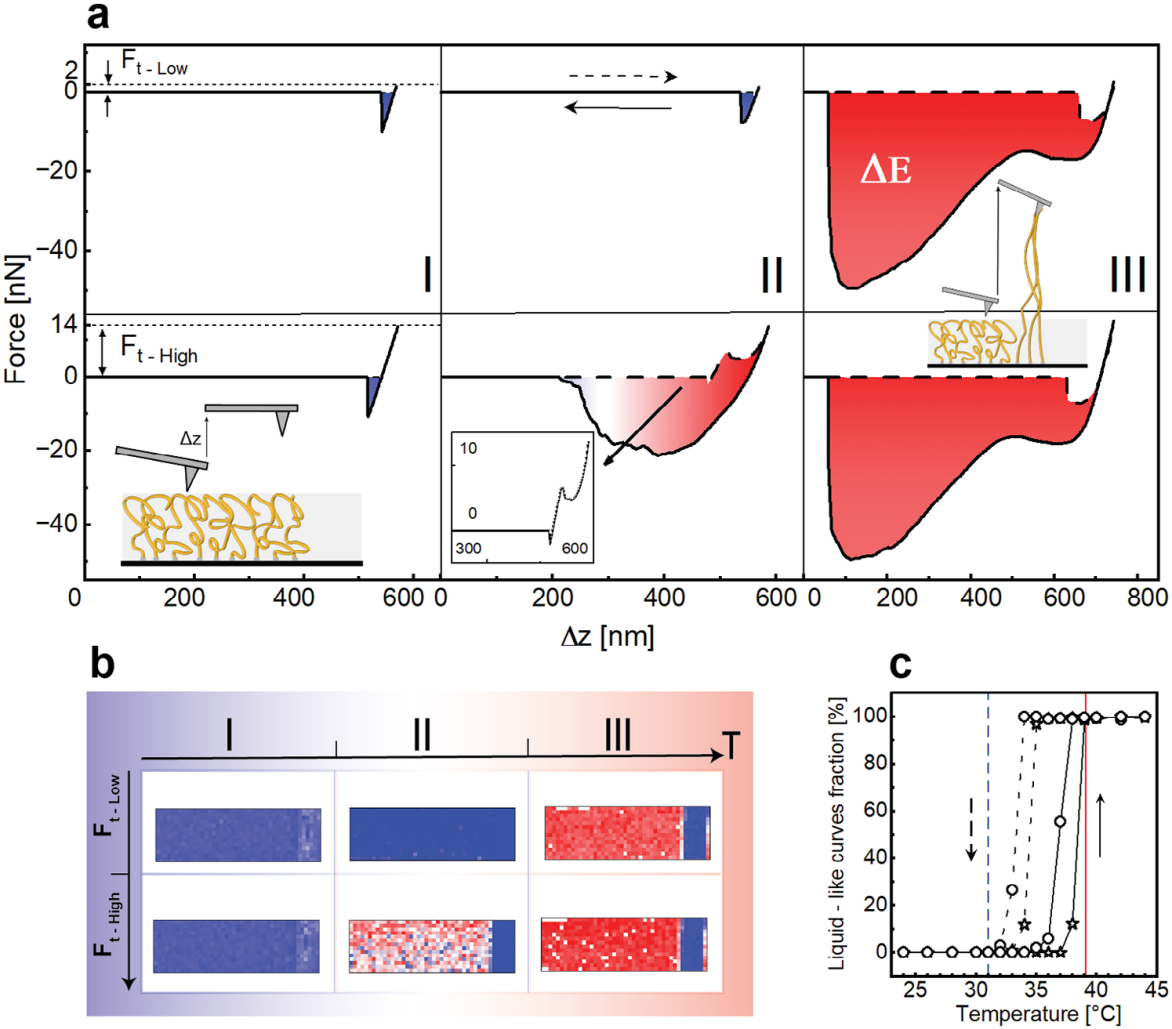
Thermal behavior of dry P18MA brushes: (AFM)‐based adhesion measurements. a) Representative force‐distance curves recorded in Force Volume mode on dry P18MA brushes in regimes I (*T* < 34 °C), II (35 °C < *T* < 38 °C), and III (*T* > 38 °C), for low (top) and high (bottom) threshold force (*F_t_*). The curves for low *F_t_* display weak solid‐like adhesion, while those for high *F_t_* display stronger liquid‐like response. Dashed and solid arrows indicate approach and withdrawal direction. Shaded areas: energy dissipation. Insets show: illustrative sketches of AFM tip and substrate interactions in regime I (left), and regime III (right); zoomed‐in approach curve for regime II at high *F_t_*. b) Energy dissipation maps derived from force volume data for low (top) and high (bottom) *F_t_* across temperature regimes I, II, and III. Color scale: regime I—*ΔE* ranges from blue (0) to red (1 fJ); regime II—from blue (0) to red (6 fJ); regime III—from blue (0) to red (20 fJ). The map covers a width of 50 µm, with the blue stripe representing the SiO_2_ wafer reference (See Figure S2a, Supporting Information for additional data). c) Fraction of liquid‐like curves vs. temperature (same data as panel a). Solid line: heating; dashed line: cooling. Stars: low *F_t_*; circles: high *F_t_*.

To understand the origin of these distinct swelling and wetting regimes, we characterize the mechanical response of dry P18MA layers using Atomic Force Microscopy (AFM) in Force Volume mode at variable temperatures. Like in the case of the wetting experiments, the AFM experiments display three distinct regimes (**Figure** **2**a), with the transition temperatures for these dry brushes being slightly higher.

Force distance curves in regime I (up to 34 °C) display a similar behavior as conventional hard solid surfaces, such as the underlying silicon wafer: upon approaching the surface, the cantilever remains undeflected until it ‘snaps to contact’ from a very small distance of a few nanometers. Upon pressing the cantilever further toward the sample, the hard repulsion of the substrate leads to a linear upward bending of the cantilever. As the cantilever motion is reversed from approach to retract at some threshold force *F_t_*, linear downward bending of the cantilever is observed indicating a finite adhesion. Upon pulling further, the adhesive force is eventually overcome and the cantilever snaps back to its undisturbed initial configuration. The distance between snap‐to‐contact and snap‐off from the surface is a few tens of nanometers for the present type of cantilevers consistent with adhesion forces of 10 nN. This qualitative behavior at low temperatures is independent of *F_t_*.

Upon entering regime II (35–38 °C for dry films) at somewhat higher temperatures, the response of the system becomes more complex and depends on the maximum applied threshold force: for low *F_t_* (2 nN in the top panel), the force curves are similar as the solid‐like behavior observed in regime I. For higher *F_t_* (14 nN bottom panel), however, approach curves become non‐monotonic and the retraction curves display an enhanced (≈20 nN) and in particular very long‐range adhesive force with an often gradual decay to zero after a few hundred nanometers of retraction.

At even higher temperatures, in regime III (>38 °C), the *F_t_*–dependence disappears again. Irrespective of *F_t_*, the approach curve is characterized by a relatively abrupt onset of an attractive force that gradually reverses into repulsion as the cantilever is pushed further toward the sample. Upon retracting the cantilever, we observe again an adhesive force of now up to ∼50 nN with a very long‐range of up to 500 nm before the tip detaches from the sample. Similar retraction curves have been reported earlier for polymer surfaces in the molten state and are attributed to bundles of mobile polymer chains that adhere to the cantilever and are gradually pulled out of the sample until they detach when more or less fully stretched.^[^ ^29^, ^30^ ^]^ In view of the melting temperature of P18MA bottle brushes,^[^ ^31^ ^]^ we interpret the nano‐mechanical response of our brush layers in regime III as liquid‐like. This is also consistent with the gradual onset of the adhesive force upon approaching the surface, which has been interpreted before by Walker et al. as a sign of liquid and highly mobile polymer molecules engulfing the tip after initial contact.^[^ ^32^ ^]^ An obvious consequence of the liquid‐like response is a dramatic increase in the energy dissipated throughout an AFM‐approach‐retract cycle at elevated temperature, as evident from the energy dissipation maps (Figure 2b). See also Figure S2b,c (Supporting Information) for additional data.

The *F_t_*_–_ dependent response in regime II suggests that the brush layers are in an intermediate configuration between the solid‐like response at low temperature and the liquid‐like response at high temperature. Specifically, the observation of a solid‐like response at low *F_t_* and a liquid‐like response at high *F_t_* suggests the presence of a thin solid layer residing like a crust on the surface of an otherwise already molten polymer layer. Gently touching the surface with the AFM tip at low *F_t_* allows to probe of the fragile solid crust, while applying higher forces breaks the crust and probes the properties of the underlying molten ‘bulk’ part of the brush layer. Accordingly, AFM measurements display low energy dissipation at low *F_t_* and high energy dissipation for high *F_t_* in regime II (Figure 2b). The idea of the force‐induced breaking of the crust is further supported by the approach curve at high *F_t_* in regime II, which initially displays a solid‐like response with a linearly increasing repulsive force until that trend is broken and the force decreases again in a manner that is comparable to the onset of the attractive forces seen in the approach curves in regime III (see bottom inset Figure 2a ‐regime II).

Analyzing the force curves from area maps such as the one shown in Figure 2b for a range of temperatures, we find that the transition from 100% solid‐like to 100% liquid‐like response typically occurs within ≈1 °C, indicating a very sharp transition (Figure 2c; see Methods section for a description of the classification scheme into solid‐ and liquid‐like response). For low *F_t_* (stars), the transition occurs ≈2 °C higher than for high *F_t_* (circles). Interpreting the first appearance of liquid‐like force curves at high *F_t_* as an indication of the onset of bulk melting and complete disappearance of solid‐like response curves at low *F_t_* as complete melting of the crust at the surface, we identify bulk and surface melting temperatures up heating of 34.5 and 38.5 °C based on the AFM measurements. Upon cooling, we observe a hysteresis in the AFM response of ≈4 °C, both for bulk and surface melting.

### 2.3 Surface Versus Bulk Melting

**Figure 3.**
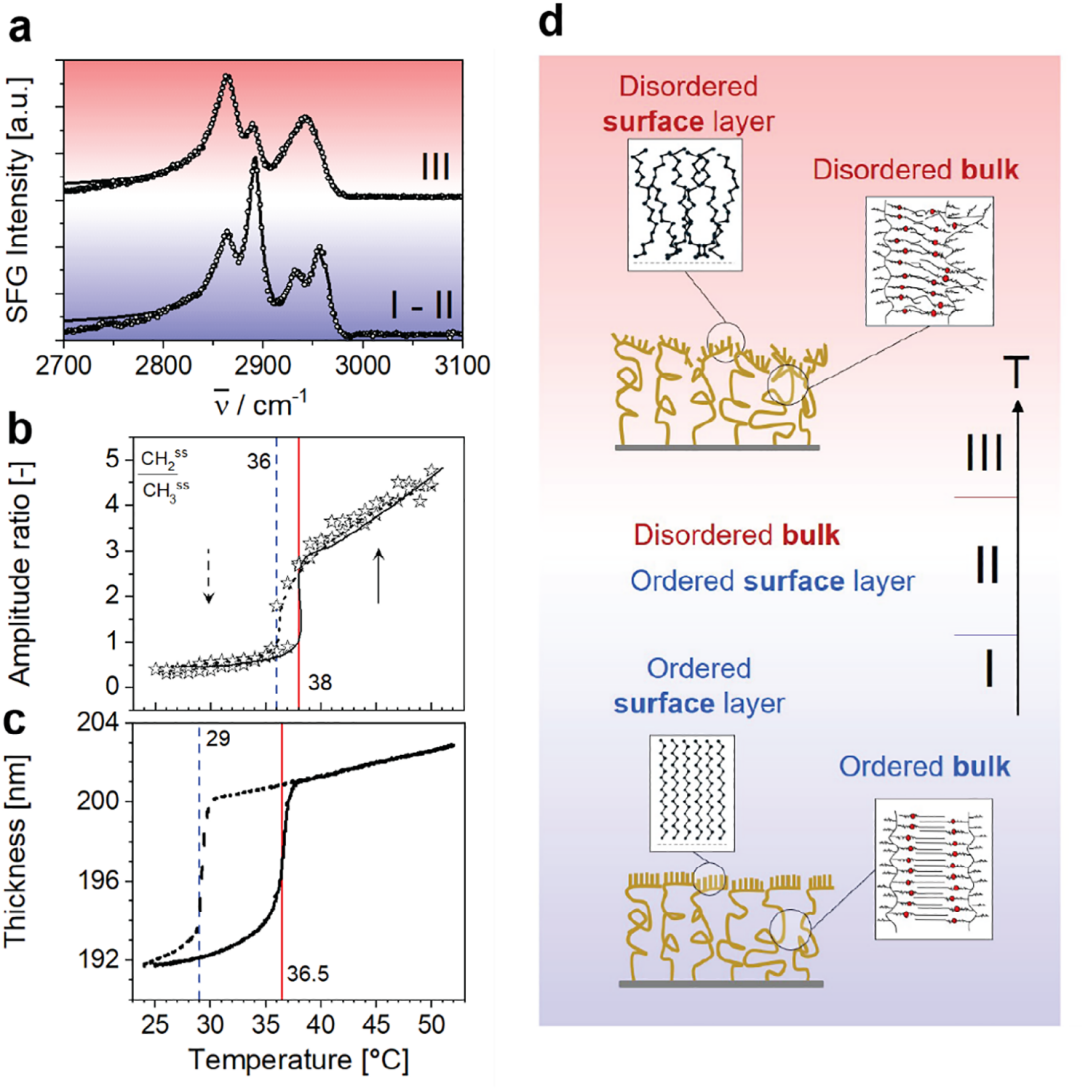
Effect of temperature at P18MA brushes/air interface. a) Representative SFG spectra of C‐H bands for dry P18MA brushes/air in regime I – II (24 °C < *T* < 38 °C; bottom) and III (*T* > 38 °C top). Open circles: experimental data points; solid lines: Lorentzian fits. b) CH_2_^ss^/ CH_3_^ss^ amplitude ratio as function of temperature. Solid line: heating; dashed line: cooling. c) Temperature‐dependent ellipsometry. Solid line: heating; dashed line: cooling. d) Illustration depicting the transition from ordered to disordered states at the P18MA brushes/air interface upon heating to 38 °C and cooling to 35.5 °C. The sketch highlights the changes in molecular orientation at the interface with temperature.

The observation of a rigid surface layer—a single crystalline monolayer formed on the surface of an isotropic liquid—echoes findings from earlier studies on pure normal alkanes and comb‐like poly(n‐alkyl acrylate) polymers.^[^ ^28^, ^33^, ^34^, ^35^ ^]^ This phenomenon contrasts typical polymer thin films where chains near the free interface experience less steric hindrance as compared to bulk polymer, resulting in a lower melting temperature.^[^ ^36^, ^37^, ^38^, ^39^ ^]^ To verify whether similar processes take place at the surface of our polymer brushes, we performed vibrational Sum‐Frequency Generation (SFG) spectroscopy at different temperatures. As explained in more detail in the Supporting Information, SFG is an inherently surface‐specific method that can interrogate the terminal layer of the P18MA brushes and is highly sensitive to breaks in centrosymmetry.^[^ ^40^, ^41^ ^]^ In situ SFG spectra (**Figure** **3**a) recorded in temperature regime I (up to 34 °C) are dominated by characteristic bands at 2863 and ≈2888 cm^−1^ which can be attributed to CH_2_ (d^+^) and CH_3_ (r^+^) symmetric stretching vibrations, whereas additional bands at ≈2928, ≈2946, and ≈2965 cm^−1^ are due to CH_2_ asymmetric stretching (d^−^) vibrations, the methyl Fermi resonance and CH_3_ asymmetric stretching (r^−^) vibrations, respectively.^[^ ^28^, ^33^, ^34^, ^35^ ^]^ Notably, at low temperatures the high SFG intensity of the r^+^ mode relative to the much lower intensity of the d^+^ mode confirms the presence of a well‐ordered layer of the octadecyl side chains at the brush surface. Previous work on alkyl chain ordering at interfaces has shown that d^+^/r^+^ intensity ratios of <0.1 are attributable to alkyl chains with a low density of *gauche* conformations, where the chains are predominately in an *all trans* state. In such cases, the d^+^ band in SFG spectra exhibits negligible intensity as the methylene groups are in a local centrosymmetric environment.^[^ ^42^, ^43^, ^44^ ^]^ Although, in temperature regime I the d^+^/r^+^ ratios are somewhat higher than 0.1, they remain characteristic for ordered alkyl chains with their terminal CH_3_ groups pointing into the vapor phase. Next, we will make use of the strong dependency of the d^+^/r^+^ ratio on the molecular order of alkyl chains at the brush‐vapor interface to examine the temperature‐dependent structure changes. Upon increasing the temperature to regime II (35 to 38 °C), the SFG spectra remain largely unaltered, indicating only minor changes in the chain ordering at the surface. However, upon entering regime III (>38 °C) the d^+^ band (CH_2_ symmetric stretch) becomes more intense than the previously dominating r^+^ band (symmetric CH_3_ stretch) at 2888 cm^−1^ (Figure 3a,b) resulting in a d^+^/r^+^ ratio of >1. The abrupt change in d^+^/r^+^ ratio at ≈38 °C indicates a dramatic loss in molecular order of the octadecyl side chains at the surface transitioning from a solid‐like, ordered state to a disordered, liquid‐like state. Consequently, this transition corresponds to a melting transition at the surface of the P18MA brushes. To precisely determine the transition temperature, we fitted the temperature‐dependent SFG spectra in Figure 3a and plotted the d^+^/r^+^ amplitude ratio in Figure 3b. The temperature of the melting transition is found to be at 38 °C (Figure 3b) and is consistent with the temperature where the AFM measurements show a completely liquid‐like response (Figure 2c). Upon cooling, the molecular order of the surface layer is restored as the evidenced by a reduction in the d^+^/r^+^ ratio to its initial value (Figure 3b). However, the freezing transition occurs at a slightly lower temperature of 35.5 °C, implying hysteresis in the surface melting/freezing transitions. See Figure S3 (Supporting Information) for the complete range of spectra during heating and cooling.

Having established the correspondence between the disappearance of order in the surface layer and the loss of solid‐like nanomechanical surface response, we still need to independently verify the onset of melting of the bulk part at a somewhat lower temperature. To this end, we analyze the variation in the thickness of our films with temperature using ellipsometry. Since the surface layer analyzed through SFG measurements is significantly thinner than the total brush layer (in the order of 1–2 nm according to studies of similar surface‐frozen layers,^[^ ^28^, ^33^, ^34^, ^35^ ^]^) any variations in the total film thickness are expected to predominantly reflect changes in the bulk of the polymer brush layer. Temperature‐dependent ellipsometry measurements indeed demonstrate that the thickness of the film expands only very gradually for low temperatures throughout regime I (up to 34 °C). Upon entering regime II ≈35 °C the expansion rate begins to increase. Finally, at temperature between 35 and 38 °C the film thickness undergoes a rather abrupt increase of ≈5%. Beyond 38 °C, a linear thermal expansion with 3.55 × 10^−4^ K^−1^ is observed (Figure 3c). Such a discontinuous increase in film thickness is characteristic for a first order melting transition rather than a continuous glass transition, which would be expected to lead to an increase in the expansion coefficient while the film thickness remains continuous.^[^ ^45^ ^]^ The observed discontinuous increase in film thickness is consistent with the semicrystalline nature of bulk P18MA, which displays the melting of the ordered nano‐domains of ordered alkyl chains.^[^ ^27^ ^]^ Upon cooling, the transition from the thicker to the thinner state takes place only ≈29 °C. This suggests that bulk crystallization requires appreciable supercooling to enable ordering of the side chains within the dense molten brush layer.

Together, the AFM, SFG, and ellipsometry measurements thus consistently lead to the following picture (Figure 3d): dry P18MA brush layers are frozen in a solidified state at low temperatures (regime I) up to the bulk melting temperature 36.5 °C. Beyond that temperature, in regime II, the bulk of the brush layer is molten while the surface layer retains its order and remains solid up to the surface melting temperature of ≈38 °C. In regime III, beyond the surface melting temperature, the entire polymer film including the surface layer is molten. These findings offer a comprehensive understanding of the thermal behavior of P18MA brush layers, emphasizing the complex interplay between surface and bulk dynamics in polymer brushes. See Figure S6 (Supporting Information) for the thermal stability of the dry brush layer after 15 consecutive heating–cooling cycles, with no evidence of thermal degradation.

### 2.4 Reconciling Melting, Swelling and Wetting Transitions

**Figure 4:**
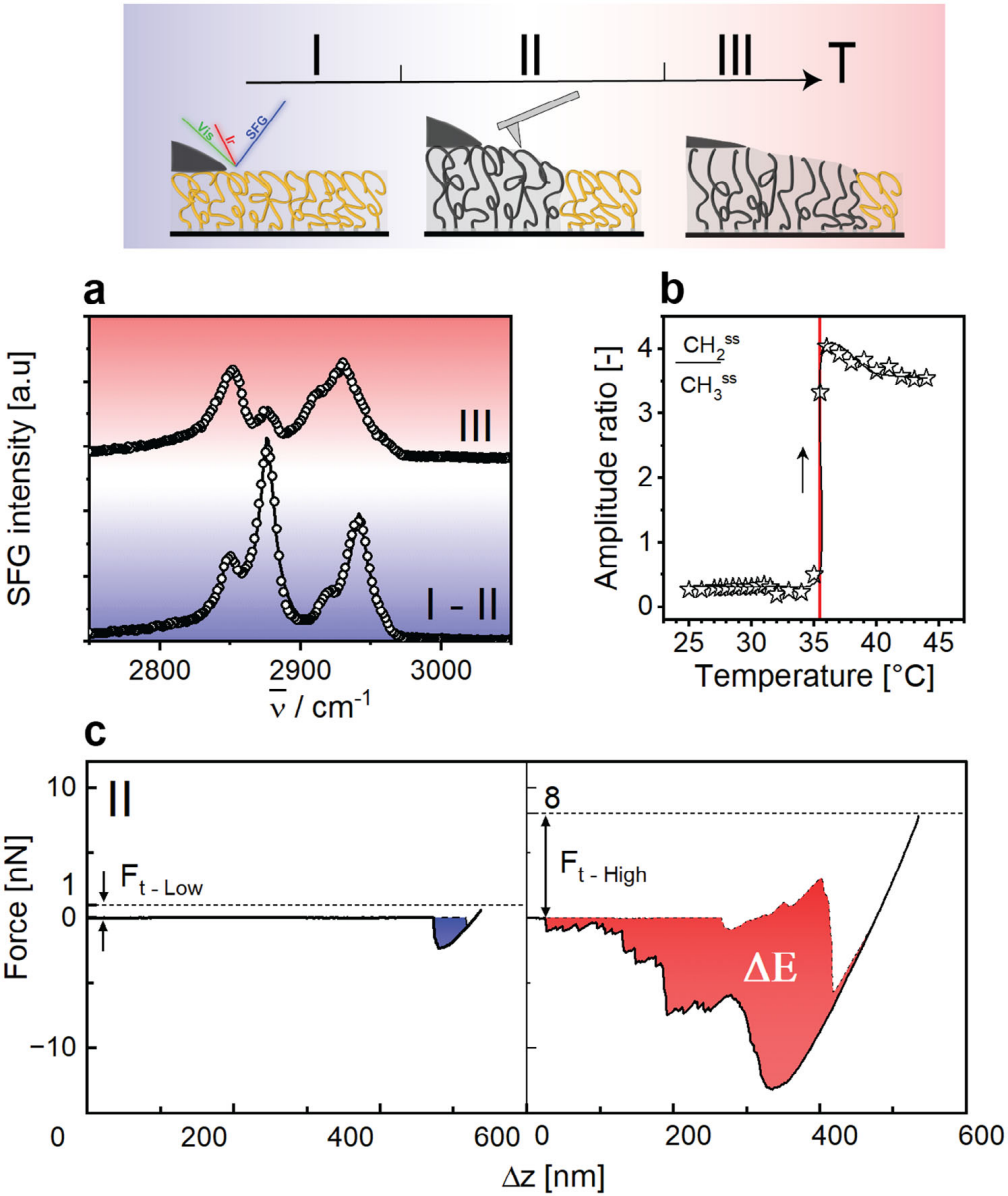
Surface ordering‐induced wetting transition. a) Representative SFG spectra of C‐H bands for P18MA brushes‐ d_34_‐hexadecane‐air interface in regime I – II (24 °C < *T* < 35.5 °C; bottom) and III (*T* > 35.5 °C top). Open circles: experimental data points; solid lines: Lorentzian fits. b) CH_2_^ss^/ CH_3_^ss^ amplitude ratio as a function of temperature during heating. c) Representative force‐distance curves recorded in Force Volume mode on P18MA brushes within the halo region in regime II (29 °C < *T* < 34 °C), for low (left) and high (right) threshold force (*F_t_*). Dashed curves: approach; solid curves: withdrawal. Shaded areas: energy dissipation.

While the existence of three different regimes in the nanoscopic properties of the dry films coincides with the existence of three different macroscopic swelling and wetting regimes, the actual transition temperatures are quite different between the dry and the wet situations. To confirm the relation between the two situations, we repeated our nanoscopic characterization experiments in the presence of a hexadecane (oil) drop. In this case, the response of the polymer brush obviously depends on the location that is probed with respect to the macroscopic oil drop on the surface. Far away from the drop, the behavior and transition temperatures of the dry film are recovered. In the vicinity of the drop, however, the situation is different. The SFG spectra (**Figure** **4**a) recorded upon focusing the laser beams adjacent to the drop display at low temperatures the same familiar pattern of a well‐ordered CH_3_‐terminated alkyl layer with some shoulders originating from CH_2_ symmetric stretch (d^+^) as in the case of a dry film (Figure 3a). As we discuss in more detail in the Supporting Information, the frequencies of the vibrational modes are slightly red‐shifted compared to dry brush, which we attribute to the interaction with the deuterated d_34_‐hexadecane with the alkyl chains of the surface layer close to the three‐phase contact line.^[^ ^46^, ^47^ ^]^ Upon increasing the temperature, the system enters regime II, i.e. the brush layer swells, and the halo forms around the drop (*T* = 29 °C, Figure 4a). This dramatic macroscopic transition leaves the SFG spectra recorded in the halo region largely unaffected throughout the entire regime II. Only upon entering regime III when the drop spreads, the SFG spectra change (*T* = 35.5 °C) and become dominated by the d^+^ mode, again indicating a disordered molten surface layer (Figure 4b; note that the position of the laser has to be laterally shifted at this temperature because of the spreading of the drop). (See Figure S4a, Supporting Information for the complete range of spectra). AFM measurements confirm this picture: in regime II, which now corresponds to the temperature range of 29 to 34 °C, the force curves display the coexistence of liquid‐like and solid‐like behavior, depending on the threshold force *F_t_* within the halo region of the partly swollen brush (Figure 4c). Farther away, a solid‐like response is observed (Figure S4b, Supporting Information), consistent with the measurements on the dry solid film in Figure 2. Hence, we conclude that the macroscopic swelling and wetting transitions are driven by the same bulk melting and surface melting transitions that we also find for the dry polymer brush layers. The primary difference is that the presence of the oil, which acts as a good solvent for the polymer, leads to a reduction of both melting transitions to somewhat lower temperatures. Such a solvent‐induced reduction of melting transitions is common for many materials and is driven by mixing entropy.^[^ ^48^ ^]^ For polymers, this effect is also known as the plasticizing effect of good solvents and it has been observed for brushes in contact with vapor as well.^[^ ^21^, ^49^ ^]^ Macroscopic wetting and swelling experiments show that the melting point depression is more pronounced for smaller alkane molecules that are better solvents than longer alkanes.^[^ ^50^, ^51^ ^]^ (Videos S1–S5, Supporting Information).

### 2.5 Thermal Activation of Frozen Polymer Brushes: Solvent Entrapment, Transport, and Vapor Sensing

**Figure 5:**
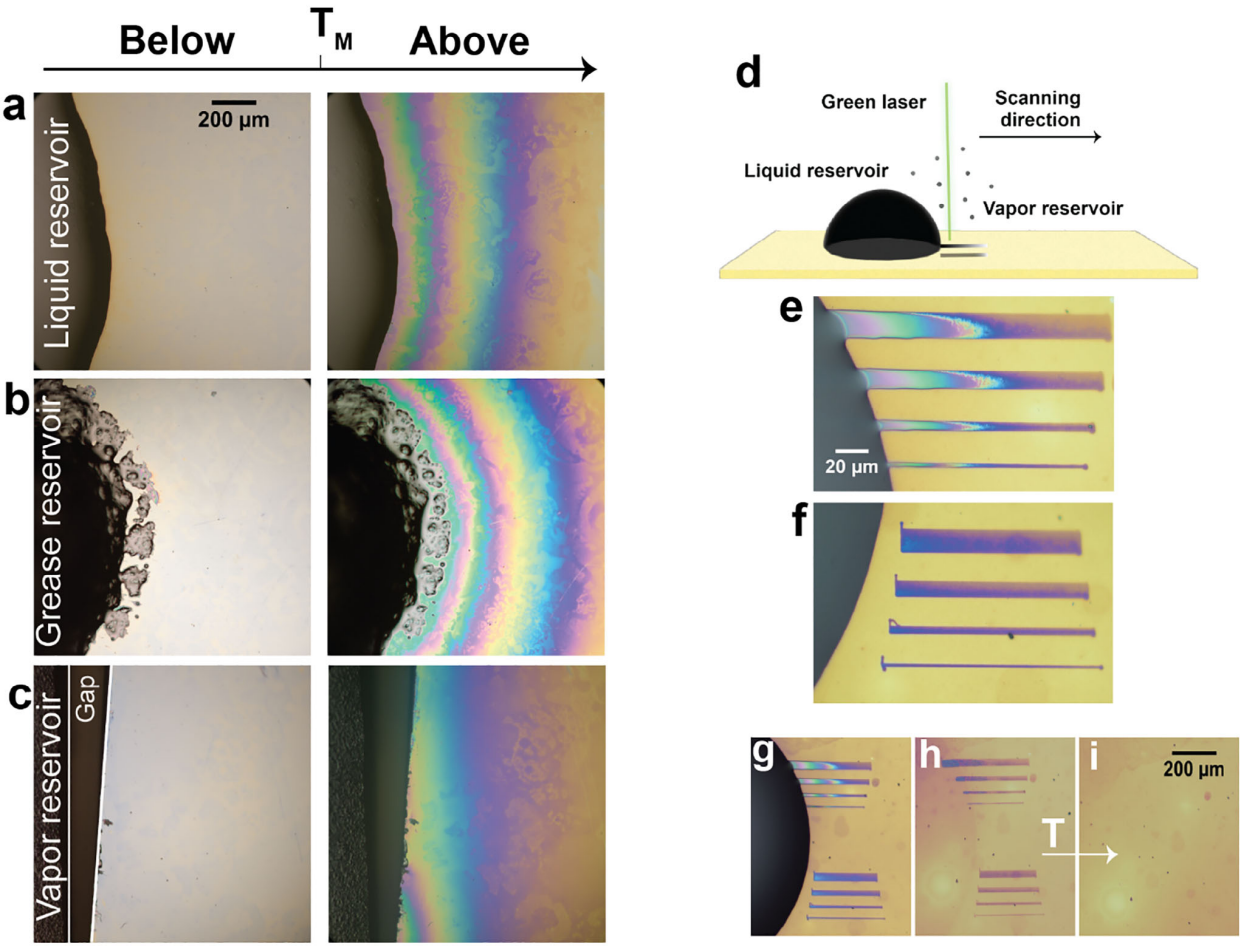
Global and laser–induced thermal activation of frozen polymer brushes. a,b,c) Optical top‐view images of P18MA brushes below and above the melting transition in the presence of: a liquid (octadecane) reservoir (a), image on the left taken after 16 h at 29 °C, image on the right taken after 7 h in the temperature range 29 °C < *T* < 36 °C; grease reservoir (b), image on the left taken after 16 h at room temperature, image on the right taken after 22 h in the temperature range 24 °C < *T* < 45 °C; vapor (hexadecane) reservoir (c), image on the left taken after 1 h at room temperature, image on the right taken after 30 min at 38 °C. The scale bar is identical for all images. d) Schematic of the experimental setup for the laser‐induced patterning. e,f) Optical images of patterned P18MA brushes displaying lines with thicknesses of 15, 10, 5, and 2 µm. The top image shows patterns created by dragging liquid hexadecane from the droplet, while the bottom image illustrates the patterning achieved by trapping vapor hexadecane. The scale bar is identical for all images. g) Zoomed out‐image of patterned P18MA, showing both patterns from liquid and vapor hexadecane. h,i) Optical images demonstrating the reversibility of the patterning process: after droplet removal with a nitrogen gun (h) and subsequent heating of the sample to 40 °C within 5 min (i). The scale bar is identical for images g‐i.

The complex swelling and wetting transitions described above emerge from the fact that the surface‐grafted P18MA polymer brush layers cannot respond to the ambient fluid at room temperature because they are arrested in a solidified semicrystalline configuration. Instead, they act like a dry frozen sponge. Only once heated above the melting temperature (*T_M_*), their characteristic ability to swell is activated. As a result, ambient fluid is absorbed and the halo of a partially swollen brush can form, as seen in regime II in Figure 1a. These observations suggest the concept of using dry frozen polymer brushes as functional coatings ‘on hold’ that can be activated on demand by stimulating the transition to the molten state. Once ‘unleashed’ in this manner, the brush layer can perform its desired function. **Figure** **5** illustrates this melting‐controlled ability to sorb and transport a solvent over macroscopic distances of hundreds of micrometers for a series of examples (Videos S6–S11, Supporting Information). In each case, the brush layer can be exposed to the fluid or vapor for many hours without swelling for temperatures below *T_M_*. As soon as *T_M_* is exceeded, fluid sorption and lateral diffusion within the brush layer begin. Fluid can be sorbed from a bulk drop (Figure 5a), from vapor (5c), or even from another ‘soft sponge’ loaded with oil, such as a commercial lubrication Li/M grease (5b).

In addition to globally heating the entire sample, we can also activate the P18MA layer locally by heating it with a focused laser. In this case, the laser beam is absorbed in the underlying silicon substrate, which is, consequently, heated and can induce local melting of the brush layer at the laser focus. In the presence of an oil drop that serves as a reservoir, the molten brush locally absorbs oil from the drop and starts to swell. By scanning the laser focus across the surface, we can write patterns of swollen polymer brush within an otherwise dry collapsed matrix with a width as low as 2 µm within 1 min (Figure 5e,f). Once the laser is deactivated or moved to a different location, the brush solidifies and entraps the sorbed fluid at the intended positions. This loading process is most efficient in the vicinity of the reservoir drop, as indicated by the more intense color and thickness gradient of the lines (Figure 5e–g). The solidified surface layer acts as a barrier layer, suppressing evaporation and enabling the preservation of a permanent pattern that (depending on the solvent) can be maintained indefinitely. Subsequent heating to a temperature above the bulk melting temperature erases the pattern and brings the P18MA layer back to its pristine original state (Figure 5h,i). (See Video S11, Supporting Information). This highlights the unique reversibility of our patterning process, a feature that distinguishes it from conventional approaches where the patterning is typically irreversible.^[^ ^52^, ^53^, ^54^ ^]^ Such reversibility enables repeated cycles of pattern formation and erasure, offering a novel and versatile approach in the field.

Figure 5f,g illustrates that fluid is not only incorporated into the brush layer by direct imbibition in contact with the fluid reservoir but also if the laser‐induced melting is initiated some distance away from the drop. In this case, the strongly negative chemical potential of oil vapor drives sorption from the vapor phase in the initially dry brush.^[^ ^55^, ^56^, ^57^, ^58^, ^59^ ^]^ While somewhat less effective than direct imbibition this process nevertheless also leads to substantial local swelling and thus a transformation of the brush layer. This sorption from the vapor phase can be further extended to use the brush layer as a sensor for solvent vapor that can be activated on demand by heating and read out at a later stage based on the color and thickness changes induced by the entrapped solvent (Figure 5c). Similarly, reversible fluid storage could be useful for entrapping and releasing specific solute molecules, as required in various drug delivery applications.^[^ ^14^, ^60^, ^61^ ^]^

## 3 Conclusion

Overall, our experiments demonstrate that polymer brushes with a sufficiently high melting temperature can be prepared in a dry, collapsed, and frozen state, in which they do not respond to ambient solvent. Only upon heating beyond *T_M_*, their ‘conventional’ responsiveness to ambient solvent is enabled, allowing for swelling, fluid transport, and entrapment, and–thanks to the independent melting transition of the surface layer–for a separate transition in wettability. This suggests a strategy to design polymer brush systems with enhanced functionality based on two key ingredients: the first one is to exploit not only the polymer‐solvent interaction but also to make explicit use of the polymer‐intrinsic melting transition as an additional external control parameter. The second one is to design the material deliberately to display a surface layer with a different (higher) melting temperature such that the bulk and the surface layer can be activated independently. In this manner, the exchange of solvent and solute molecules with the environment can be triggered by melting the surface layer, while transport of already sorbed fluid within the bulk of the brush layer is still possible at intermediate temperatures. The present P18MA material displays this property by virtue of the surface freezing effect; other brush systems may require a dedicated block architecture to achieve this functionality. Notably, the laser–induced patterning method introduced here offers a significant advantage by enabling reversible and rewritable laser writing, allowing precise control over droplet manipulation. We anticipate that this generic approach will enable a new class of highly flexible polymer brush systems, with potential applications spanning across sensing, controlled lubricant transport, color‐based displays, and drug delivery.

## 4 Experimental Section

### Materials

Native oxide silicon wafers (100 ± 0.5mm diameter, 525 ± 25 µm thickness, boron‐doped, <100> orientation, 5–10 Ωcm, OKMETIC) were cut into 1 × 1 cm pieces and used as a substrate for polymer brush growth. The polymerization was performed in 5mL (40 × 20 mm, fisherbrand) snap cap glass vials. (3‐aminopropyl) triethoxysilane (APTES, 99%, Sigma Aldrich), triethylamine (TEA, >99.5%, Sigma Aldrich), α‐bromoisobutyryl bromide (BiBB, >98.0% TCI chemicals), L‐ascorbic acid (AA, >99%, Sigma Aldrich), N,N,N′,N″,N″‐pentamethyldi‐ethyleentriamine (PMDETA, 99%, Sigma Aldrich), CuCl_2_ (97%, Sigma Aldrich), octadecyl methacrylate (18MA, 97%, TCI chemicals), ethanol (absolute ≥99%, Fisher), toluene (HPLC grade, VWR chemicals), N,N‐dimethylformamide (DMF, 99.8%, Thermo Scientific), deuterated hexadecane‐d_34_ (99% atom D, abcr), n‐octadecane (99%, Sigma Aldrich), Li/M grease (lithium thickener, mineral base oil with base oil viscosity 100cSt at 40 °C), n‐hexadecane (99%, Sigma Aldrich) were purchased and used without further purification.

### Surface Anchor and Radical Initiator Functionalization

The silicon substrates were sonicated in ethanol for 1 min, and rinsed with demi‐water and ethanol before being subjected to ozone plasma treatment for 15 min. The activated wafer pieces were immediately placed into a desiccator around a petri‐dish containing (3‐aminopropyl) triethoxysilane (0.1 mL, 0.43 mmol, APTES). The desiccator was then evacuated for 15 min and subsequently closed, allowing for overnight vapor deposition. The amine‐terminated substrates were rinsed twice with ethanol and demi‐water and blown dry under a nitrogen stream. Along with a stirring bar, the samples were placed face‐up into a reaction vessel. In an Erlenmeyer flask, cooled toluene (100 mL) and triethylamine (1.12 mL, 8.05 mmol TEA) were combined. While stirring vigorously, α‐bromoisobutyryl bromide (1 mL, 8.09 mmol BiBB) was added dropwise. This solution was transferred to the aforementioned reaction vessel containing the substrates and allowed to react for 3 h while stirring. The substrates were rinsed twice with toluene, ethanol, and demi‐water, and dried under a nitrogen stream.

### Brush Polymerization

P18MA brushes were synthesized via Activators ReGenerated by Electron Transfer‐Atom Transfer Radical Polymerization (ARGET‐ATRP) in individual 5mL snap cap glass vials filled with 7 mL of solution. Three stock solutions were prepared: 1) L‐ascorbic acid (74 mg, 420 µmol, AA) in DMF (10 mL), 2) CuCl_2_ (28 mg, 210 µmol) in DMF (10ml) sonicated to dissolve the CuCl_2,_ with N,N,N′,N″,N″‐pentamethyldi‐ethyleentriamine (0.1ml, PMDETA), and 3) octadecyl methacrylate (11.9 g, 35.1 mmol, 18MA) in DMF (10 mL) dissolved at 40 °C.

Into each vial, 2.7mL of the AA solution 1) was injected, followed by 0.45ml of the catalyst solution 2), followed by 3.75mL of the DMF/18MA 3) solution. The molar ratio of AA:CuCl_2_:PMDETA:18MA in the reaction mixture was 12:1:2.3:590.

A functionalized wafer was placed face‐up into the solution, the vial sealed, and left to react for the desired time. Afterward, the polymer brushes were rinsed twice with toluene, ethanol, and demi‐water, and blown dry under a nitrogen stream.

### Macroscopic Spreading Behavior

The temperature‐dependent spreading behavior of *n*‐alkanes on P18MA brushes was studied using optical top‐view imaging. Images were captured with an upright Nikon Eclipse L150 microscope, equipped with a 10× objective (working distance: 5 mm) and a Basler a2A5328‐15ucBAS color camera. The samples were placed on a heated element (Dimension Icon heater/cooler) connected to a Thermal Applications Controller (TAC) under ambient conditions. The temperature was controlled and varied between 23 °C (room temperature) and 40 °C. Qualitatively similar behavior was observed for more than 10 samples from different synthesis batches.

### Contact Angle

Static contact angle measurements were performed on an optical contact angle device (DSA25, Krüss, Germany). Heating was performed by the same Dimension Icon heating stage as mentioned above, in open air. A hexadecane droplet was deposited on the brush surface at the desired temperature, and the contact angle was recorded every 2 s by fitting the contour with the Young‐Laplace equation. For each temperature, the presented contact angles were averaged over 100 measurements on a single sample, taken after the droplet had equilibrated for at least 1 min. Qualitatively similar behavior was observed for 3 samples from different synthesis batches.

### Brush Height Profile

Brush swelling profiles were obtained through custom written colorimetric analysis. The ideal spectral reflectance of an air–brush layer with variable thicknesses–silicon substrate system was simulated, which was converted to color coordinates by the transfer matrix method.^[^ ^62^, ^63^, ^64^ ^]^ Using a chromaticity matching function, allows us to simulate the observed brush color for every brush thickness, resulting in a ‘standard color map’. Experimentally, the actual colors of the brush layers were recorded using an optical microscope and matched to this standard color map to determine the thickness for each pixel. The color matching was performed using the deltaE CIE76 method, with colors represented in the CIELAB color space. The known brush dry thickness from ellipsometry was used as a reference.

### Atomic Force Microscopy (AFM)

AFM in tapping mode was employed to characterize the dry thickness, topography, and adhesion properties of the brush layers both in dry and in the presence of oil. All measurements were performed using a Bruker Icon (Santa Barbara, CA, USA) with silicon probes (tip radius r < 8 nm, spring constant k ≈ 0.6 Nm^−1^, HQ: NSC36/Cr‐Au BS, MikroMasch). To establish a reference, the brush layer was locally removed using a sharp needle to create a cross‐section. Topography and dry thickness were analyzed in tapping mode under ambient conditions (Figure S5, Supporting Information).

Force–distance curves (FDCs) were recorded in force volume mode with a constant approach and retraction rate of 1 Hz and applied threshold forces (*F_t_*) ranging from 2 to 14 nN. The experiments were conducted under controlled temperature conditions, varied between 23 °C (room temperature) and 44 °C, over a 50 µm scanning area, collecting ≈800 force‐distance curves for both low and high threshold forces. Data analysis was performed using custom Python scripts. Retraction curves were categorized into liquid‐like and solid‐like behaviors based on piezo displacement (Δz): curves with Δz > 80 nm were classified as liquid‐like, while those with Δz ≤ 80 nm were considered solid‐like. For wet adhesion measurements, an oil droplet was placed on the brush layer and the sample was heated until a visible halo formed (Regime II). Measurements were conducted at a constant approach and retraction rate of 1 Hz, with applied threshold forces (*F_t_*) ranging from 1 to 8 nN. Heating was performed by the same Dimension Icon heating stage described above, operating in open air. The presented force‐distance curves were single representative curves extracted from one force‐volume measurement, and consistent with measurements on 4 different samples (not shown).

### Sum‐Frequency Generation (SFG)

A broadband SFG spectrometer that was described in detail elsewhere^[^ ^65^ ^]^ was employed. Briefly, the pulse energy from a Spectra‐Physics Soltice Ace amplifier system was split into two beams used to pump an optical parameter amplifier Topas Prime (Light Conversion) with a subsequent unit for noncollinear difference‐frequency generation and to create an etalon‐filtered narrowband visible pulse at 804.1 nm with a bandwidth of <5 cm^−1^. The broadband IR beam with a center‐frequency of 2900 cm^−1^, a bandwidth of >300 cm^−1^ and 10 µJ pulse energy was overlapped at the sample surface with the visible narrowband pulse with ≈2 ps pulse duration and 20 µJ pulse energy. The beam diameters at the short axis of the elliptically shaped focus of both beams were at the sample position 260 and 530 µm for the IR and VIS beams, respectively. The SFG beam reflected from the sample surface was guided to a Kymera spectrograph (Andor) equipped with a 1200 lines mm^−1^ grating and subsequently detected using an electron‐multiplied charge‐couple device (EMCCD) (Newton, Andor).

SFG spectra of the brush layers, both in dry conditions and in the presence of oil, were recorded with a total acquisition time of 60 s using s‐, s‐, and p‐polarized (ssp) SFG, VIS, and IR beams, respectively. All spectra were normalized to the non‐resonant signal of an air‐plasma‐cleaned polycrystalline Au film deposited on a Si wafer. Samples were placed in the spectrometer's sample compartment, which was purged with dry air to maintain a relative humidity of <5%. Temperature‐dependent studies were conducted by first equilibrating the samples at 25 °C using a custom‐built sample holder with resistive heating capability. The temperature was then gradually increased at a rate of ≈0.3 °C min^−1^, with a PT100 sensor in close contact with both the sample and the holder to monitor the temperature. For SFG measurements on dry brushes, the temperature was raised to a maximum of 80 °C, with SFG spectra recorded throughout the heating process. This was followed by a cooling phase, during which the sample temperature was reduced to 25 °C at a rate of ≈0.1 °C min^−1^, with additional in situ SFG spectra recorded. For measurements in the presence of oil, SFG spectra were recorded near the three‐phase contact line of a droplet of deuterated hexadecane‐d_34_ (0.8 µL). In these measurements, the temperature was increased from 25 to 45 °C. To analyze the SFG spectra in greater detail, the data were fitted with Lorentzian functions as described in the Supporting Information (Table S1, Supporting Information). Each presented SFG spectrum represents a single measurement. The observed trend was consistent between measurements on 5 different samples (not shown).

### Ellipsometry

Individual thickness measurements were performed on a M2000‐X spectroscopic ellipsometer (J.A. Woolam controlled by completeEASE software, operating in the wavelength range of 300–1000nm, with 5s sampling time at three angles of incidence (65, 70, and 75°). For variable temperature measurements, a temperature‐controlled cell (HFS600, Linkam Scientific Instr.) with windows fixed at an angle of incidence of 70° was used with 1s acquisition time. The data was fitted using an optical model composed of an infinitely thick Si substrate, a 2.5nm thick native SiO_2_ layer, and a Cauchy layer fitting the first and second order parameters. A pre‐determined fixed angle offset was used based on a reference silicon wafer. When using the Heated Cell, a delta offset was variably fitted to account for window effects from the chamber. In the measurement of Figure 3, a continuous temperature ramp of 0.33 °C min^−1^ was performed from 25‐52‐25 °C, twice. The second heating and cooling cycle was shown in the graph, to exclude solvent‐induced memory effects. The data presented corresponds to a single measurement. Qualitatively similar behavior was observed on 3 more samples from different synthesis batches for at least 2 heating cycles.

### Global and Laser‐Induced Thermal Activation of Frozen Polymer Brushes

Global thermal activation experiments were conducted using three types of reservoirs: liquid oil (n‐octadecane), Li/M grease, and vapor oil (n‐hexadecane). Imaging was performed with an upright Nikon Eclipse L150 microscope equipped with a 10× objective (working distance: 5 mm) and a Basler a2A5328‐15ucBAS color camera. Heating was achieved using the Dimension Icon heating stage (as previously described), operating in open air for liquid oil and grease reservoirs and in a closed cell for the vapor reservoir.

For the liquid oil reservoir, solid octadecane was deposited onto the dry brush surface, and the temperature was gradually increased to its melting point (29 °C). Subsequently, the temperature was varied between 29 and 35 °C (Regime II). For the grease reservoir, a grease patch was applied to the dry brush surface, and the temperature was controlled from room temperature to 45 °C. In the vapor reservoir setup, liquid hexadecane was deposited onto a silicon wafer placed at a fixed distance from the dry brush layer, with the system confined in a closed cell. The temperature was increased from room temperature to 38 °C.

Laser‐induced thermal activation experiments were conducted with a liquid oil (hexadecane) reservoir in open air. Line patterns were created using a confocal Witec Alpha 300R microscope with a 532 nm laser. The laser power was set to 35 mW, with an integration time of 0.01 s. Each pattern was scanned 15 times with 100 points per line. A consistent response was observed in 10 additional samples from different synthesis batches.

### Statistical Analysis

In this work, data (Figure 1b,c; Figures S2b,c and S5, Supporting Information) were reported as the mean ± standard deviation (SD). The contact angles in Figure 1b are averaged over 100 measurements, while the swelling profile in Figure 1c is based on the average of 24 lines. In Figure S2b,c (Supporting Information), adhesion and energy dissipation values for each temperature were determined from the average of 800 force‐distance curves. For Figure S5 (Supporting Information), the thickness was calculated as the average over 10 lines.

## Conflict of Interest

The authors declare no conflict of interest.

## Author Contributions

L.B. and F.M. conceived the study and designed the experiments. L.B., S.R., B.S., E.L., and F.N. conducted the experiments and analyzed the data. L.B. and F.M. drafted the manuscript with feedback from all authors. B.B. and S.d.B. provided additional contributions to the revision and editing of the manuscript. All authors discussed the results and approved the final version of the manuscript.

## Supporting information



Supporting Information

Supplemental Video 1

Supplemental Video 2

Supplemental Video 3

Supplemental Video 4

Supplemental Video 5

Supplemental Video 6

Supplemental Video 7

Supplemental Video 8

Supplemental Video 9

Supplemental Video 10

Supplemental Video 11

Supplemental Video 12

## Data Availability

The data that support the findings of this study are available from the corresponding author upon reasonable request.
