# Supplementary material for: Thermally Activated Swelling and Wetting Transition of Frozen Polymer Brushes:a New Concept for Surface Functionalization
Source: Adv Mater. 2025 Apr 14;37(26):2502173. doi: 10.1002/adma.202502173 (PMC12232226; doi:10.1002/adma.202502173)
Supplement: Supplementary file 1 — Supporting Information [file ADMA-37-2502173-s007.docx]

**Supporting information**

**Thermally Activated Swelling and Wetting Transition of Frozen Polymer Brushes:**

**a New Concept for Surface Functionalization**

*Luciana Buonaiuto, Sander Reuvekamp, Billura Shakhayeva_,_ Enqing Liu, Franziska Neuhaus, Björn Braunschweig, Sissi de Beer, Frieder Mugele^*^*


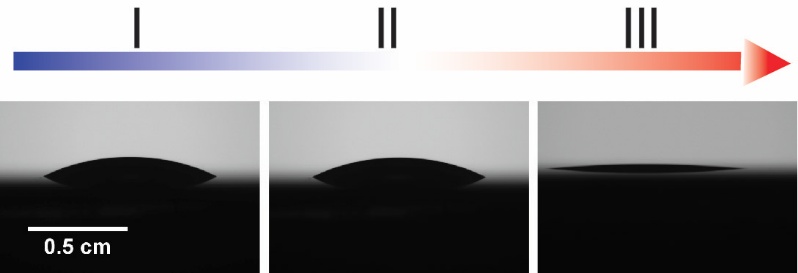


**Figure S1**. Temperature – driven spreading dynamics of hexadecane on P18MA brushes. Side view images of a hexadecane droplet on P18MA brushes. Left: regime I (*T* < 29 °C): partially wetting drop θ = 26° on solid dry brush; regime II (29 °C < *T* < 33 °C): partially wetting drop θ = 26° surrounded by halo of swollen brush. Right: regime III (*T* > 33 °C): spreading drop θ < 5° on swollen brush.

**
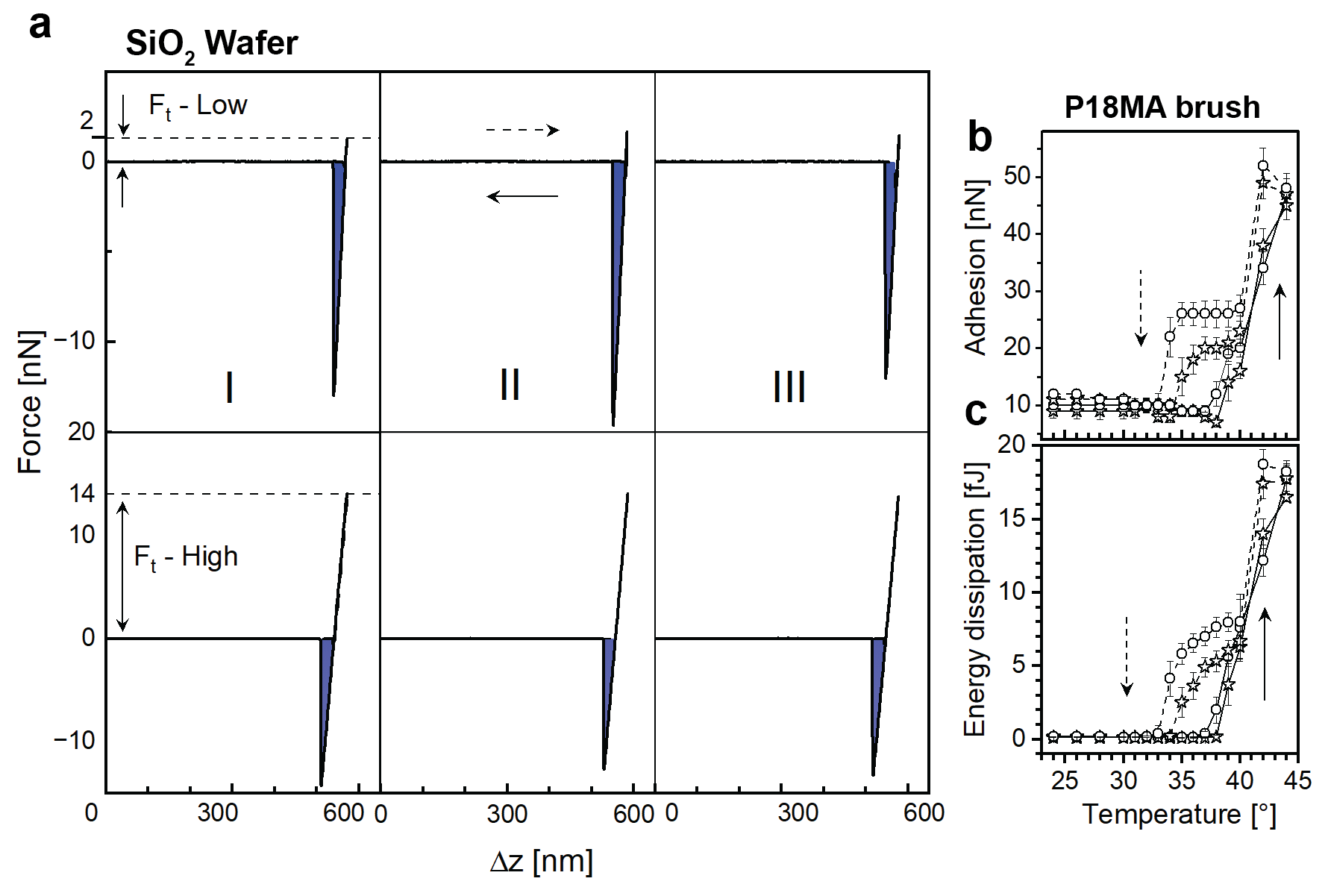
Figure S2.** AFM – based adhesion measurements. a) Representative force-distance curves recorded in Force Volume mode on the SiO_2_ wafer in regimes I (*T* < 34 °C), II (35 °C < *T* < 38 °C), and III (*T* > 38 °C), for low (top) and high (bottom) threshold force (*F_t_*). Dashed and solid arrow indicate approach and withdrawal direction. Shaded areas: energy dissipation. b) Adhesion force vs. temperature. Solid line: heating; dashed line: cooling. Stars: low *F_t_*; circles: high *F_t_*. c) Energy dissipation vs. temperature. Solid line: heating; dashed line: cooling. Stars: low *F_t_*; circles: high *F_t_*.

**Basic principles of sum-frequency generation (SFG) spectroscopy and fit procedure**

Sum-frequency generation (SFG) spectroscopy is a powerful tool for probing molecular structures at surfaces and interfaces.^[1-3]^ In SFG spectroscopy, a broadband infrared (IR) laser pulse (ω_IR_) is overlapped in time and space with a fixed-frequency visible (VIS) laser pulse ($\omega_{VIS}$), resulting in the generation of a third beam at the sum-frequency ($\omega_{SFG}=\omega_{IR}+\omega_{VIS}$). The intensity of the SFG signal depends on the effective second-order electric susceptibility $\chi^{(2)},$ which can be expressed as a combination of resonant and non-resonant components. The following equation describes the SFG intensity in terms of an effective $\chi^{(2)}$ :^[1-3]^

$$I_{SF}\propto\left| \chi_{NR}^{\left( 2 \right)}+\sum_{q} \frac{A_{q} e^{i\varphi_{q}}}{\omega_{IR}-\omega_{q}+i\Gamma_{q}} \right|^{2}I_{IR}I_{VIS} (S1)$$

where $\chi_{NR}^{\left( 2 \right)}$ represents a frequency-independent non-resonant contribution from the interface, and the Lorentzian terms capture the resonant contributions due to molecular vibrational modes at the interface. Here, $A_{q}$ is the amplitude of the q-th vibrational mode, $\omega_{q}$ the eigenfrequency of the q^th^ vibrational mode, $\Gamma_{q}$ its homogeneous linewidth and $\varphi_{q}$ the phase of the vibrational mode. The amplitude $A_{q}=N\langle\beta_{q}^{\left( 2 \right)}\rangle$ is directly proportional to the density N of interfacial molecules and the orientational average $\left\langle\ldots\right\rangle$ of the molecular hyperpolarizability $\beta_{q}^{\left( 2 \right)}$. This orientational average is significant because it vanishes in centrosymmetric materials, such as bulk liquids, materials with diamond structure like Si, gases, and amorphous materials, resulting in negligible SFG signals from these bulk phases. However, at surfaces and interfaces, the symmetry is necessarily broken, which leads to nonzero components of $\chi^{(2)}$ arising from interfacial molecules. This symmetry-breaking at interfaces makes SFG spectroscopy intrinsically interface-specific and sensitive to changes in the molecular ordering of interfacial species.


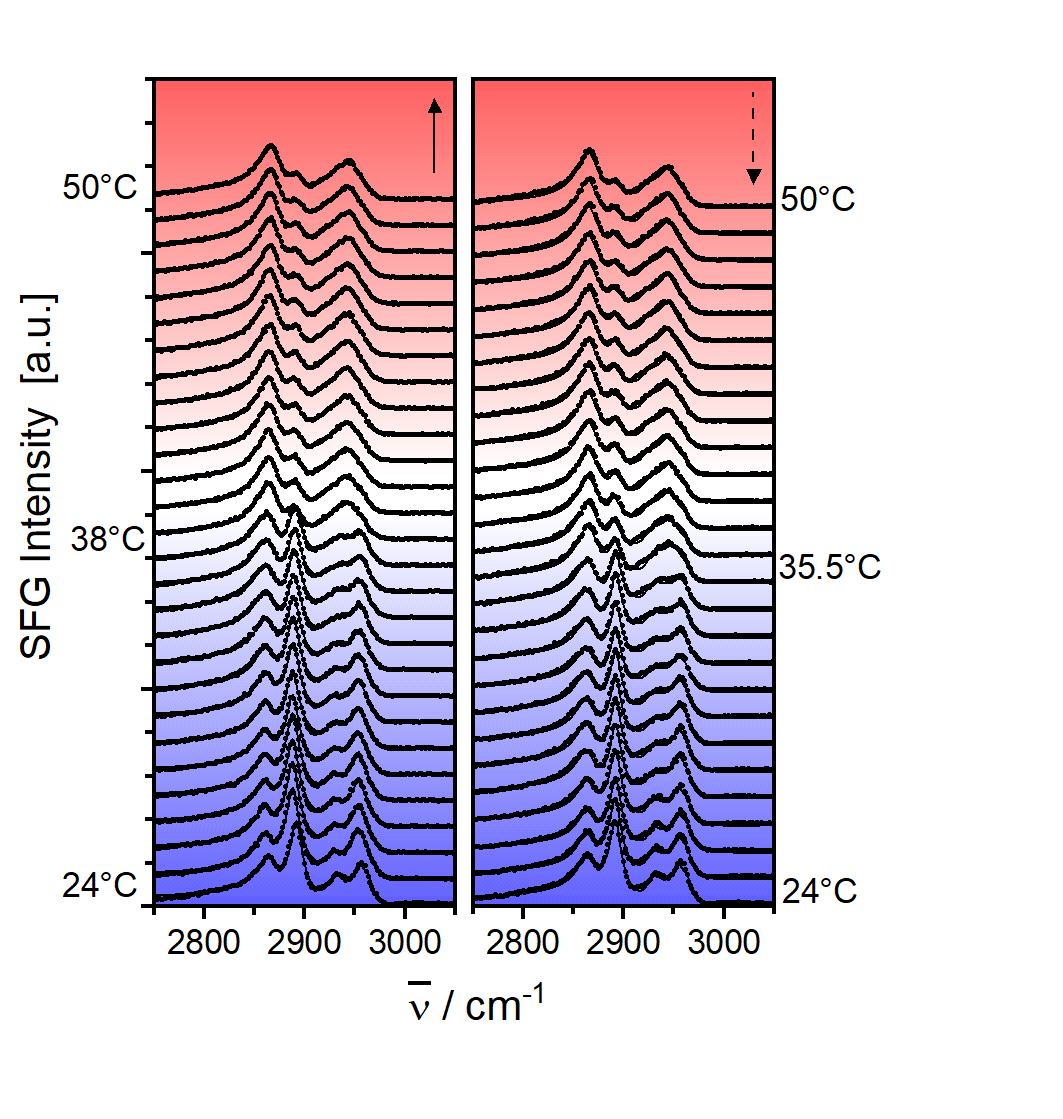


**Figure S3.**Effect of temperature at P18MA brushes/air interface. SFG spectra of C-H bands for dry P18MA brushes/air during heating (left) and cooling (right). The spectra were recorded from room temperature up to 50 °C. Open circles: experimental data points; solid lines: Lorentzian fits. Figures S3 and S4 present a series of SFG spectra from P18MA brushes in contact with air only (Figure S3) as well as in contact with deuterated hexadecane (d_34_-HD) as a function of temperature which was varied between 25 °C and 50 °C as indicated in the figures. Note that the spectra are discussed and analyzed in detail in the main text. In order to be able to perform a more quantitative analysis we have fitted the SFG spectra in Figures S3 and S4 using model functions according to eqn. (S1) using a self-written MATLAB code. Representative fit parameter of SFG spectra recorded at temperatures of 25 °C and 50 °C which is well above the polymer phase transition are shown in Table S1.

**Table S1**. Representative fit parameters from the analysis of SFG spectra of P18MA brushes in different environments as noted in the table.

| P18MA – air interface | | | | | | P18MA - d_34_-hexadecane – air interface | |
| --- | --- | --- | --- | --- | --- | --- | --- |
| **Band**  **assignment** | | 25 [°C] | 95% confidence interval | 50 [°C] | 95% confidence interval | 25 [°C] | 95% confidence interval |
|  | $\chi_{\mathrm{Nres}}^{(2)}$ | 0.12 | 0.01 | 0.12 | 0.01 | 0.05 | 0.02 |
| **d^+^** | A_q_ / a.u | 0.17 | 0.04 | 1.5 | 0.03 | 0.8 | 0.07 |
|  | $\Gamma_{q}$*/* cm^-1^ | 9.8 | 0.97 | 10 | 0.88 | 5.9 | 1.05 |
|  | *φ / π* | 0.72 | 0.03 | 0.93 | 0.04 | 0.4 | 0.4 |
|  | $\omega_{q}$ */ cm^-1^* | 2863 | 0.89 | 2863 | 0.8 | 2848 | 0.6 |
| **r^+^** | $A_{q}$ */ a.u* | 1.73 | 0.04 | 0.5 | 0.03 | 3.03 | 0.08 |
|  | $\Gamma_{q}$ */cm^-1^* | 5.3 | 0.26 | 5.3 | 0.23 | 4.2 | 0.23 |
|  | *φ / π* | 1 | N.A. | 1 | N.A. | 1 | N.A. |
|  | $\omega_{q}$ */ cm^-1^* | 2888 | 0.27 | 2892 | 0.25 | 2876 | 0.2 |
| **d^-^** | $A_{q}$ */ a.u* | 0.5 | 0.04 | 0.5 | 0.03 | 0.6 | 0.07 |
|  | $\Gamma_{q}$ */cm^-1^* | 8.7 | 1.43 | 10 | 1.35 | 11.5 | 3.2 |
|  | *φ / π* | 1 | N.A. | 1 | N.A. | 0.85 | N.A. |
|  | $\omega_{q}$ */ cm^-1^* | 2928 | 1 | 2928 | 0.96 | 2913 | 1.9 |
| $\boldsymbol{CH}_{\boldsymbol{3}}^{\boldsymbol{FR}}$ | $A_{q}$ */ a.u* | 1.2 | 0.05 | 1.07 | 0.57 | 2.3 | 0.14 |
|  | $\Gamma_{q}$ */cm^-1^* | 5.5 | 0.88 | 9.5 | 0.87 | 4.7 | 0.49 |
|  | *φ / π* | 0 | N.A. | 0 | N.A. | 0 | N.A. |
|  | $\omega_{q}$ */ cm^-1^* | 2946 | 0.89 | 2945 | 0.9 | 2941 | 0.5 |
| **r^-^** | $A_{q}$ */ a.u* | 0.4 | 0.1 | 0.2 | 0.11 | 0.4 | 0.18 |
|  | $\Gamma_{q}$ */cm^-1^* | 5 | 1 | 6.6 | 0.95 | 6.3 | 2.9 |
|  | *φ / π* | 1 | N.A. | 1 | N.A. | 1 | 0.1 |
|  | $\omega_{q}$ */ cm^-1^* | 2965 | 1.15 | 2965 | 1.42 | 2965 | 2.5 |


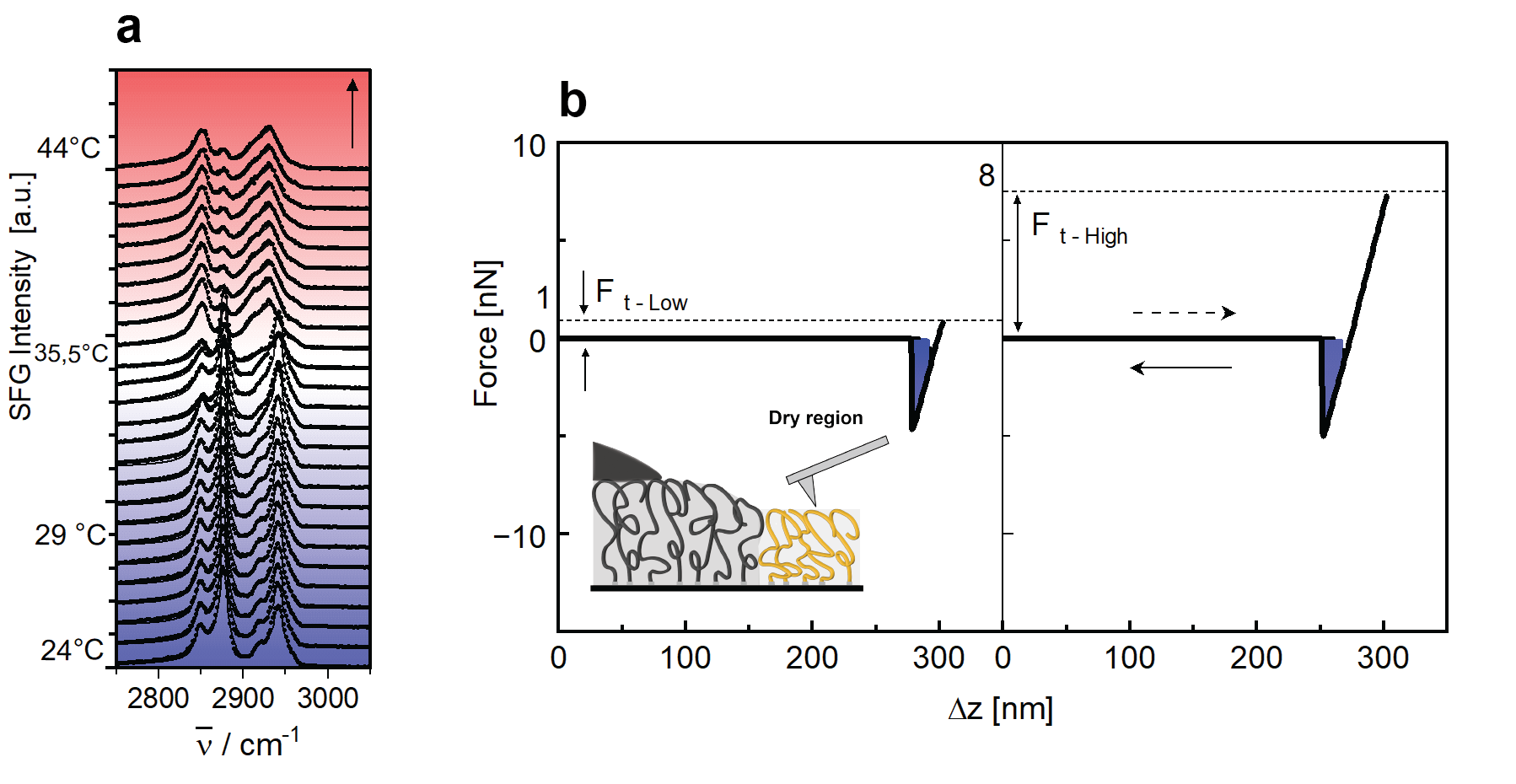


**Figure S4.** Reconciling melting and wetting transition. a) SFG spectra of C-H bands at P18MA brushes- d_34_-hexadecane-air interface during heating. The spectra were recorded from room temperature up to 50 °C. Circles: experimental data points; solid lines: Lorentzian fits. b) Representative force-distance curves recorded in Force Volume mode on P18MA brushes in the dry region in regime II (29 °C < *T* < 34 °C), for low (left) and high (right) threshold force (*F_t_*). Dashed curves: approach; solid curves: withdrawal. Shaded areas: energy dissipation.


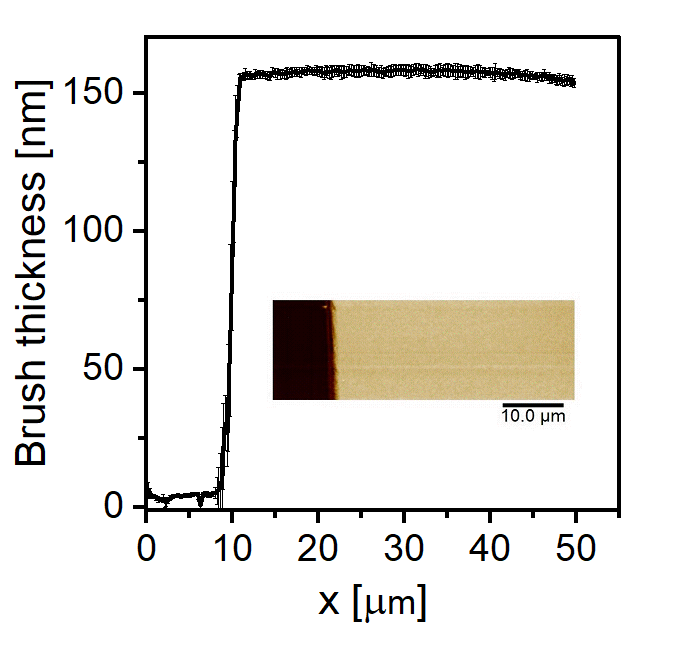


**Figure S5.** Thickness profile and topography of P18MA brushes via AFM. Thickness profile of P18MA brushes measured via AFM in tapping mode. The inset shows the corresponding topographic image, highlighting the boundary between the SiO₂ substrate (dark stripe, left) and the P18MA brush (light stripe, right), with the brush layer exhibiting smooth and uniform morphology.

**
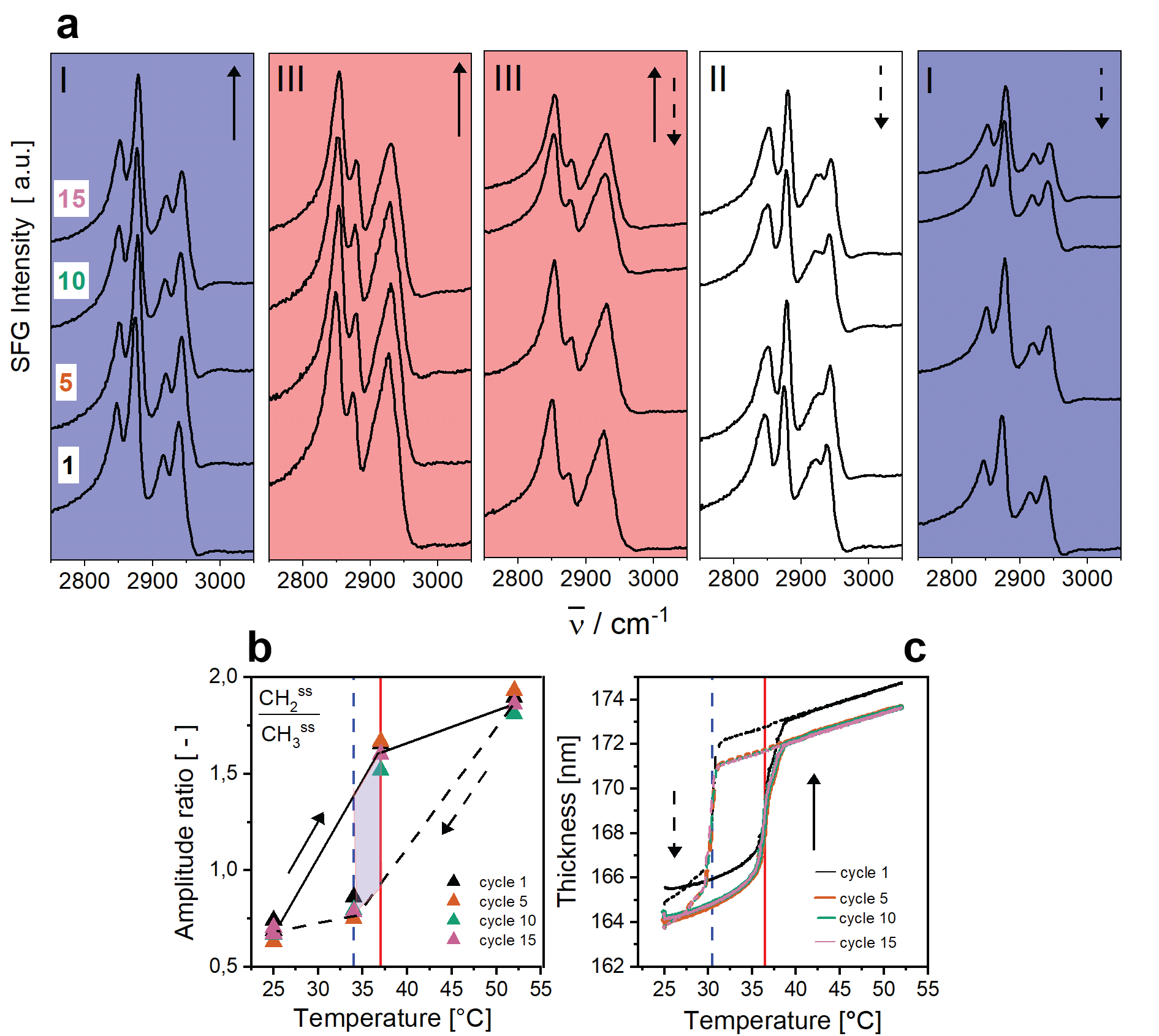
**

**Figure S6. Thermal Stability of the Dry P18MA Brush Layer.** A total of 15 heating–cooling cycles were performed on the dry P18MA sample. SFG and ellipsometry data were recorded during cycles 1,5,10 and 15 to assess the stability and reproducibility of the thermal response. No thermal degradation is observed after 15 heating-cooling cycles with melting/crystallization occurring at the same temperature. a) SFG spectra of C-H bands for dry P18MA brushes/air at 24 °C (Regime I), at 37 °C (Regime III), at 52 °C (Regime III), at 34 °C (Regime II) and at 24 °C (Regime I) during heating and subsequent cooling (left to right) for cycle 1, 5, 10, 15 (bottom to top). b) CH₂ₛₛ/CH₃ₛₛ SFG amplitude ratio as a function of temperature. Solid line: heating; dashed line: cooling. Black triangles: cycle 1; Orange triangles: cycle 5, Green triangles: cycle 10, and Pink triangles: cycles 15. c) Temperature-dependent ellipsometry measurements. Note: the difference in brush thickness between cycle 1 and 5 is attributed to annealing effects, after which no thickness reduction is observed. Furthermore, after sample washing the same thickness was measured (not shown). Cycles 1, 5, 10 and 15 are measured with a temperature ramp rate of 0.33 °C min^-1^. Solid line: heating; dashed line: cooling. Black line: cycle 1; Orange line: cycle 2, Green line: cycle 10, and Pink line: cycle 15.


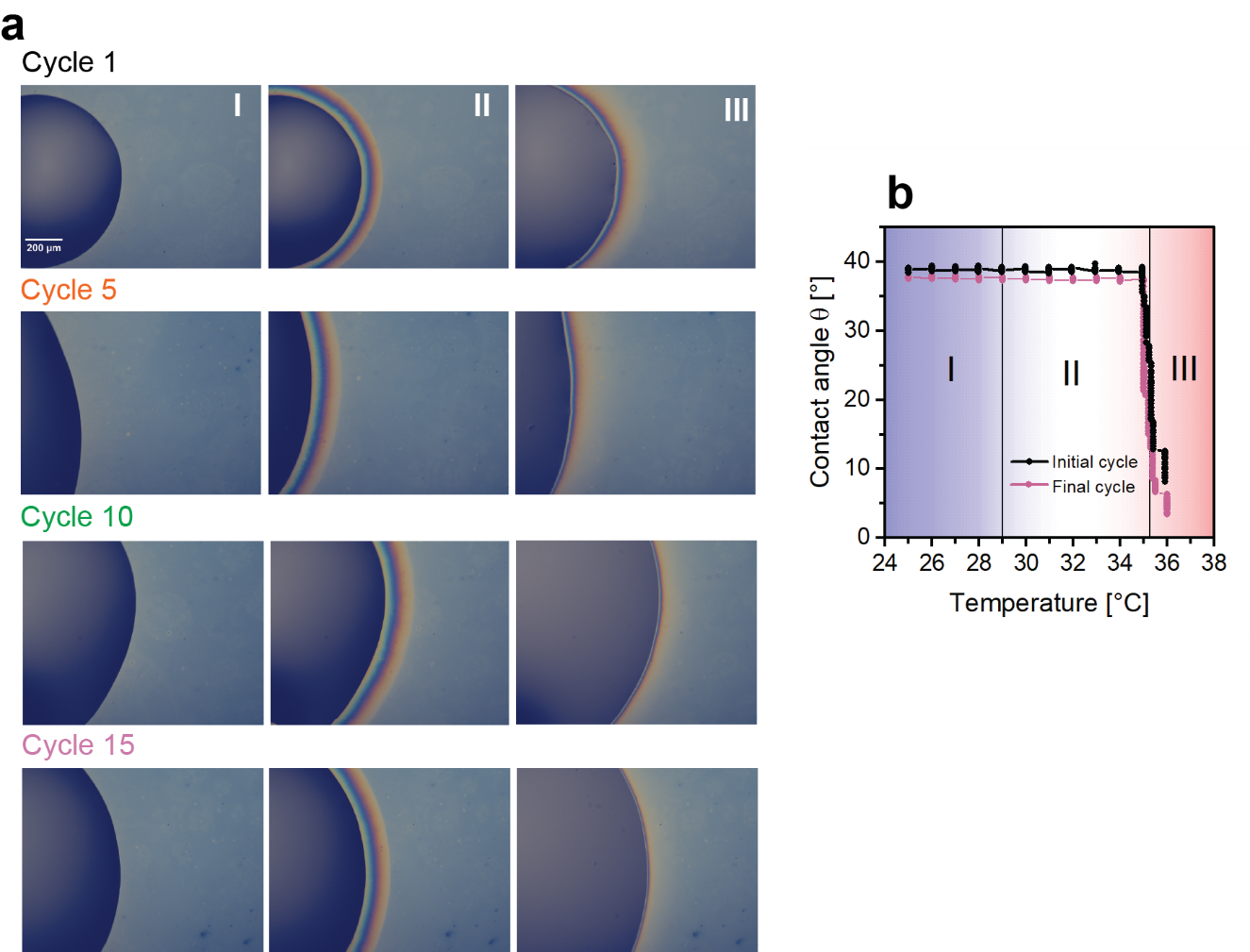


**Figure S7.** Thermal Stability of P18MA Brush Layer in the presence of a hexadecane drop. A total of 15 heating–cooling cycles were performed on the P18MA brush layer in the presence of a hexadecane drop. Optical top view images were recorded during heating from cycle 1 to 15. a) Optical top-view images of a hexadecane droplet (gray) on P18MA brushes (blue). Left to right: regime I (T < 29 °C): partially wetting drop (θ = 39°) on solid dry brush; regime II (29°C < T < 33°C): partially wetting drop (θ = 39°) surrounded by halo of swollen brush; regime III (T > 33 °C): spreading drop (θ < 5°) on swollen brush. Images are shown for cycle 1, 5, 10, and 15 (top to bottom) b) Hexadecane contact angle versus temperature from side view images illustrating the invariance of the contact angle upon swelling (regime II) and the wetting transition upon entering regime III. The contact angle was measured at 0.5fps while heating the sample with 1 °C/min, until fully spread. Black circles: wetting transition of hexadecane on the fresh P18MA sample; Pink circles: wetting transition of hexadecane after approximately 15 heating–cooling cycles in presence of hexadecane.

**Videos description**

All movies were recorded with an upright Nikon Eclipse L150 microscope equipped with a 10× objective (working distance: 5 mm) and a Basler a2A5328-15ucBAS color camera. Each image is 1.45 x 1.26mm in size.

1. Dodecane (C_12_H_26_) droplet (gray) on P18MA brushes (yellow). Spreading droplet on swollen brush at room temperature. Observation time: ~3 minutes.
2. Tetradecane (C_14_H_30_) droplet (gray) on P18MA brushes (yellow). 24 °C < *T* < 28 °C : Partially wetting droplet surrounded by halo of swollen brush; *T* > 28 °C: spreading droplet on swollen brush. Observation time: ~32 minutes.
3. Hexadecane (C_16_H_34_) droplet (gray) on P18MA brushes (yellow). Regime I (*T* < 29 °C): partially wetting droplet on solid dry brush; regime II (29 °C < *T* < 33 °C): partially wetting droplet surrounded by halo of swollen brush; regime III (*T* > 33 °C): spreading droplet on swollen brush. Observation time: ~2 hours. Note: to capture the spreading process, the field of view was adjusted in the last few minutes.
4. Solid octadecane (C_18_H_38_) (black) on P18MA brushes (yellow). 29 °C: Octadecane melting transition. Regime I (29 °C < *T* < 32 °C): partially wetting droplet on solid dry brush; regime II (32 °C < *T* < 37 °C): partially wetting droplet surrounded by halo of swollen brush; regime III (*T* > 37 °C): spreading droplet on swollen brush. Observation time: ~2 hours. Note: to capture the spreading process, the field of view was adjusted in the last few minutes.
5. Solid eicosane (C_20_H_42_) (gray) on P18MA brushes (yellow). 33 °C < *T* < 38 °C: Eicosane melting transition. *T* > 38 °C: Spreading droplet on swollen brush. Observation time: ~1 hour. Note: to capture the spreading process, the field of view was adjusted in the last few minutes.
6. Solid octadecane (C_18_H_38_) (gray) on P18MA brushes (yellow). At 29 °C, the melting transition of octadecane occurs. Liquid octadecane is maintained on the brushes at 29 °C (below the brush melting transition) for ~16 hours.
7. Solid octadecane (C_18_H_38_) (gray) on P18MA brushes (yellow). At 29 °C, the melting transition of octadecane occurs. Liquid octadecane on P18MA brush within the temperature range 29 °C < *T* < 36 °C; (above the brush melting transition) for ~7 hours.
8. Li/M grease (gray) on P18MA brushes (yellow) maintained below the brush melting transition (room temperature) for ~16 hours.
9. Li/M grease (gray) on P18MA brushes (yellow) within the temperature range: 24 °C < *T* < 45 °C (above the brush melting transition) for ~22 hours.
10. Hexadecane (C_16_H_34_) (gray) on a SiO₂ wafer (right side, vapor reservoir) adjacent to P18MA brushes (yellow, left side). The system is confined in a closed cell and maintained below the brush melting transition at room temperature for ~1 hour.
11. Hexadecane (C_16_H_34_) (gray) on a SiO₂ wafer (right side, vapor reservoir) adjacent to P18MA brushes (yellow, left side). The system is confined in a closed cell and maintained above the brush melting transition within the temperature range 24 °C < T < 38 °C for ~30 minutes.
12. Residual lines due to the laser – induced thermal activation of P18MA brushes after hexadecane droplet removal with nitrogen gun. The temperature is ramped from 24 °C to 40 °C (above the brush melting transition) within ~5 minutes.

**Materials**

n-hexadecane (99%, Sigma Aldrich), deuterated hexadecane-d_34_ (99% atom D, abcr), n-dodecane (>99%, Sigma Aldrich), n-tetradecane (>99%, Thermo Scientific), n-octadecane (99%, Sigma Aldrich), n-eicosane (99% Sigma Aldrich), Li/M grease (lithium thickener, mineral base oil with base oil viscosity 100cSt at 40 °C), were purchased and used without further purification.

**References**

[1] Y. R. Shen, *J. Phys. Chem. C* **2012**, 116, 15505.

[2] A. G. Lambert, P. B. Davies, D. J. Neivandt, *Appl. Spectrosc. Rev.* **2005**, 40, 103.

[3] C. Zhang, *Appl Spectrosc* **2017**, 71, 1717.
